# Predictors of cardiovascular disease in asthma and chronic obstructive pulmonary disease

**DOI:** 10.1186/2049-6958-8-58

**Published:** 2013-09-03

**Authors:** Michela Bellocchia, Monica Masoero, Antonio Ciuffreda, Silvia Croce, Arianna Vaudano, Roberto Torchio, Monica Boita, Caterina Bucca

**Affiliations:** 1 Department of Medical Sciences, University of Turin, Via Genova 3, 10126 Turin, Italy; 2 Cardiorespiratory Pathophysiology Department, AOU S, Luigi, Gonzole Region 10, 10043 Orbassano, Turin, Italy

**Keywords:** Airway obstruction, Asthma, Cardiovascular disease, COPD, Pressure overload, Volume overload

## Abstract

**Background:**

Cardiovascular disease (CVD) is a common comorbidity in patients with chronic airway obstruction, and is associated with systemic inflammation and airway obstruction. The aim of this study was to evaluate the predictors of CVD in two different conditions causing chronic airway obstruction, asthma and COPD.

**Methods:**

Lung function tests, clinical and echocardiographic data were assessed in 229 consecutive patients, 100 with asthma and 129 with COPD. CVD was classified into: pressure overload (PO) and volume overload (VO). Sub-analysis of patients with ischemic heart disease (IHD) and pulmonary hypertension (PH) was also performed.

**Results:**

CVD was found in 185 patients (81%: 51% COPD and 30% asthmatics) and consisted of PO in 42% and of VO in 38% patients. COPD patients, as compared to asthmatics, had older age, more severe airway obstruction, higher prevalence of males, of smokers, and of CVD (91% vs 68%), either PO (46% vs 38%) or VO (45% vs 30%). CVD was associated with older age and more severe airway obstruction both in asthma and COPD. In the overall patients the predictive factors of CVD were age, COPD, and male sex; those of PO were COPD, BMI, VC, FEV_1_ and MEF_50_ and those of VO were age, VC and MEF_50_. In asthma, the predictors of CVD were VC, FEV_1_, FEV_1_ /VC%, and PaO_2_, those of PO were VC, FEV_1_ and FEV_1_ /VC%, while for VO there was no predictor. In COPD the predictors of CVD were age, GOLD class and sex, those of VO age, VC and MEF_50_, and that of PO was BMI. Sub-analysis showed that IHD was predicted by COPD, age, BMI and FEV_1_, while PH (found only in 25 COPD patients), was predicted by VO (present in 80% of the patients) and FEV_1_. In subjects aged 65 years or more the prevalence of CVD, PO and VO was similar in asthmatic and COPD patients, but COPD patients had higher prevalence of males, smokers, IHD, PH, lower FEV_1_ and higher CRP.

**Conclusions:**

The results of this study indicate that cardiovascular diseases are frequent in patients with chronic obstructive disorders, particularly in COPD patients. The strongest predictors of CVD are age and airway obstruction. COPD patients have higher prevalence of ischemic heart disease and pulmonary hypertension. In the elderly the prevalence of PO and VO in asthma and COPD patients is similar.

## Background

Asthma and chronic obstructive pulmonary disease (COPD) are the most common airway disorders with a high morbidity and mortality [1,2]. According to the Global Initiative for Asthma (GINA), approximately 300 millions people suffer from asthma [1] with a prevalence ranging from 1 to 18%. COPD prevalence is around 6%, but data are highly variable, depending on survey methods and diagnostic criteria [3].

Both asthma and COPD are characterized by inflammation, which in asthma, particularly the eosinophilic phenotype, is predominantly confined to the airway, while in COPD the inflammation may spill-over from the lungs to the systemic circulation and initiate systemic inflammation, as a consequence of exposure to indoor or outdoor air pollution, cigarette smoke, or diesel exhaust fumes [4]. A recent observation indicates that patients with neutrophilic asthma often have systemic inflammation [5]. Elevated serum levels of reactive C-reactive protein (CRP) are considered markers of systemic inflammation either in COPD or in asthma [5,6]. Systemic inflammation may be pathogenically related to many of the comorbidities seen in chronic obstructive airway diseases, including cardiovascular disease (CVD). Cardiovascular complications in COPD have been attributed to the systemic effects of smoking [7]. Several observations suggest that reduced pulmonary function, no matter what cause, is associated with increases in myocardial infarction and arrhythmia [8-11]. Forced expiratory volume in one second (FEV_1_) is ranked second to smoking and above blood pressure and cholesterol as a predictor of all-cause and cardiovascular mortality [12]. In this regard, it has been suggested that a reduction in FEV_1_ combined with smoking history better predicts cardiovascular mortality than cholesterol [13].

The aim of this study was to evaluate prevalence and predictors of CVD in two common airway obstructive diseases with quite different phenotypes, asthma and COPD.

## Methods

Consecutive adults outpatients with asthma and COPD, diagnosed according to international guidelines [1,2], were recruited from those attending the Respiratory Pathophysiology clinic of the University Hospital San Giovanni, Turin, Italy, between January 2011 and June 2012. Inclusion criteria were: age over 40 years, no acute exacerbation of COPD and asthma in the last two months, no active pulmonary tuberculosis or other clinically relevant lung disease. Patients aged below 40 years were excluded because in this age range the risk of COPD is low [14].

### Study design

Patients underwent recording of demographic data including age, full smoking history (current, former and never-smokers), recording of symptoms and medication use, clinical examination, assessment of lung function tests, arterial blood gas analysis and venous blood sampling for serum determination of CRP. Body mass index was calculated on the basis of height and weight (BMI) [15]. Asthma and COPD severity were classified according to GINA [1] and GOLD [2] criteria, respectively. Among COPD patients, those with predominant chronic bronchitis and predominant emphysema were identified.

Coexistent cardiovascular disease was assessed on the basis of history, clinical and echocardiographic data. CVD included: prior myocardial infarction and cardiovascular accidents, documented ischaemic heart disease, pulmonary or systemic arterial hypertension, heart valve disease, echocardiographic diagnosis of pulmonary hypertension.

According to the criteria of the American Heart Association [16], heart disease was classified as follows:

– pressure overload (PO), causing diastolic dysfunction with preserved systolic function, including all the conditions causing concentric left ventricle hypertrophy, such as systemic arterial hypertension and aortic valve disease

– volume overload (VO), including all the conditions causing left ventricle dilatation and systolic dysfunction, such as mitral or aortic incompetence and ischemic heart disease.

Sub-analysis was done on patients with ischemic heart disease (IHD) and pulmonary hypertension (PH).

Lung function tests were measured using the Baires System (Biomedin, Padua, Italy). The values of vital capacity (VC), forced expiratory volume in one second (FEV_1_), and their percentage ratio (FEV_1_/VC%), and maximum expiratory flow at mid VC (MEF_50_) were computed and expressed as percentage of the predicted value, according to the European Respiratory Society guidelines [17].

Arterial blood gases, that is oxygen and carbon dioxide partial pressures (PaO_2_ and PaCO_2_ respectively) were measured using the analyzer GEM 4000 PREMIERE (Instrumentation Laboratory Lexington USA).

### Statistical analysis

Data were analyzed using the SPSS software package, version 20.0 (SPSS Inc., Chicago, IL, USA) for Windows. Discrete variables are presented as counts and percentages. Continuous variables are presented as means ± SEM, as appropriate. Comparisons between asthmatics and COPD patients, and between patients with predominant chronic bronchitis and predominant emphysema were performed by the unpaired Student's *t*-test. Comparisons among CVD, PO and VO patients with univariate ANOVA. Nominal variables were compared with the Fisher's exact test and Pearson’s *χ*^2^. A stepwise backward selection procedure was used to evaluate factors influent on heart disease, using a linear regression models. The models had as dependent variables CVD, PO or VO and as independent predictors: disease (asthma or COPD), age, sex, BMI, smoking habits, GINA or GOLD class, prebronchodilator lung function tests, PaO_2_, CRP. The same models were used to evaluate predictors for asthma and COPD separately and for the sub-analysis of patients with IHD or PH.

Statistical significance was assumed at p < 0.05.

## Results

The subjects enrolled were 100 asthmatic patients and 129 COPD patients. CVD was found in 185 patients (81%), and consisted of pressure overload in 97 (42%) and of volume overload in 88 (38%). IHD was found in 58 patients (25%), 8 asthmatics and 50 COPD, and PH in 25 (11%), all with COPD. The general characteristics of asthmatic and COPD patients are compared in Table 1. The COPD group was older, and had higher prevalence of men and of both present and past smokers, more severe airway obstruction and higher prevalence of heart disease, including PO, VO, IHD and PH. Moreover, COPD patients had higher CRP values than asthmatics, suggestive of systemic inflammation. No significant difference in PaO_2_ and PaCO_2_ was found between the two groups.

**Table 1:** Comparison between general characteristics of patients with asthma and with COPD

	**ASTHMA N. 100**	**COPD N. 129**	**P**
Age, years	59 ± 1.1	69 ± 0.9	< 0.0001
BMI	25.6 ± 0.5	26.2 ± 0.45	0.010
Male, n (%)	22 (22.0)	78 (60.5)	< 0.0001
Smokers			
current, n (%)	10 (10)	72 (55.8)	< 0.0001
past, n (%)	8 (8)	42 (32.6)	< 0.0001
Atopy,n (%)	65 (73.1)	10 (7.7)	< 0.0001
Cardiovascular disease, n (%)	68 (68)	117 (90.7)	0.00002
Pressure overload, n (%)	38 (38.0)	59 (45.7)	NS
Volume overload, n (%)	30 (30.0)	58 (45.0)	0.021
Subgroup IHD, n (%)	8 (8.0)	50 (38.8)	< 0.001
Subgroup PH, n (%)	0 (0.0)	25 (19.4)	< 0.001
VC*, % predicted	88.9 ± 1.7	83.6 ± 1.8	0.033
FEV_1_*, % predicted	78.0 ± 2.0	64.6 ± 1.9	< 0.0001
FEV_1_/VC* %	65.7 ± 1.3	57.1 ± 1.3	< 0.0001
MEF_50_*, % predicted	41.8 ± 2.4	29.5 ± 2.1	< 0.0001
PaO_2_ , mmHg	74.5 ± 1.6	71.4 ± 1.0	NS
PaCO_2_ , mmHg	38.4 ± 0.5	39.8 ± 0.5	NS
CRP, mg/ml	3.4 ± 0.4	7.6 ± 0.7	< 0.0001
Beta adrenergic therapy, n (%)	94 (94.9)	97 (75.2)	0.0001
Corticosteroid therapy, n (%)	95 (96)	90 (69.8)	< 0.0001
Anticholinergic therapy, n (%)	22 (22)	76 (58.9)	<0.0001

Value recorded before bronchodilator.

The comparisons among subgroups with no cardiovascular disease (No CVD), with PO and VO in the overall patients, in asthma and in COPD are reported in the Tables 2, 3 and 4.

**Table 2:** Comparisons among patients without cardiovascular disease (no CVD), with pressure overload (PO) and volume overload (VO) in the overall study population

**ASTHMA + COPD**	**no CVD N. 44**	**PO N. 97**	**VO N. 88**	**Univariate ANOVA**	**no CVD vs PO**	**no CVD vs VO**	**PO vs VO**
Age, years	53.3 ± 1.3	64.3 ± 1.0	69.1 ± 1.1	< 0.0001	< 0.0001	< 0.0001	0.002
Male, n (%)	18 (40.9)	43 (44.3)	39 (44.3)	NS	NS	NS	NS
BMI	24.7 ± 0.7	26.9 ± 0.6	25.5 ± 0.5	0.038	0.028	NS	NS
VC, % pred.	93.4 ± 2.2	85.1 ± 1.7	83.5 ± 2.4	0.011	0.005	0.006	NS
FEV_1,_ % pred.	82.6 ± 2.5	70.7 ± 2.1	64.8 ± 2.4	< 0.0001	0.001	< 0.0001	NS
FEV_1_/VC %	67.6 ± 1.7	61.5 ± 1.5	56.7 ± 1.6	< 0.0001	0.015	< 0.0001	0.032
MEF_50,_ % pred	49.5 ± 4.1	35.8 ± 2.3	25.8 ± 2.3	< 0.0001	0.002	< 0.0001	0.003
CRP, mg/ml	3.1 ± 0.6	5.9± 0.6	7.3 ± 1.0	0.011	0.009	0.007	NS

*NS* not statistically significant.

**Table 3:** Comparisons among patients without cardiovascular disease (no CVD), with pressure overload (PO) and volume overload (VO) in asthma

**ASTHMA**	**no CVD N. 32**	**PO N. 38**	**VO N. 30**	**Univariate ANOVA**	**no CVD vs PO**	**no CVD vs VO**	**PO vs VO**
Age, years	51.3 ± 1.4	60.7 ± 1.6	63.1 ± 2.1	< 0.0001	< 0.0001	< 0.0001	NS
Male, n (%)	9 (28.1)	6 (15.8)	7 (23.3)	NS	NS	NS	NS
BMI	24.5 ± 0.8	26.5 ± 0.9	25.7 ± 0.7	NS	NS	NS	NS
VC, % pred.	94.0 ± 2.8	84.7 ± 2.2	89.0 ± 3.7	NS	0.011	NS	NS
FEV_1,_ % pred.	85.4 ± 2.9	76.7 ± 2.9	73.8 ± 4.5	0.029	0.041	0.032	NS
FEV_1_/VC %	68.9 ± 1.7	65.7 ± 2.0	61.2 ± 2.9	0.042	NS	0.010	NS
MEF_50,_ % pred	51.3 ± 4.2	37.8 ± 3.5	35.8 ± 4.5	0.017	0.016	0.015	NS
CRP, mg/ml	2.5 ± 0.5	4.1 ± 0.8	3.5 ± 0.8	NS	NS	NS	NS

**Table 4:** Comparisons among patients without cardiovascular disease (no CVD), with pressure overload (PO) and volume overload (VO) in COPD

**COPD**	**no CVD N. 12**	**PO N. 59**	**VO N. 58**	**Univariate ANOVA**	**no CVD vs PO**	**no CVD vs VO**	**PO vs VO**
Age, years	58.5 ± 2.6	66.7 ± 1.3	72.4 ± 1.0	< 0.0001	0.012	< 0.0001	0.001
Male, n (%)	9 (75)	37 (62.7)	32 (55.2)	NS	NS	NS	NS
BMI	25.4 ± 1.5	27.1 ± 0.7	25.4 ± 0.6	NS	NS	NS	NS
VC, % pred.	91.8 ± 3.12	85.3 ± 2.4	80.0 ± 2.9	NS	NS	NS	NS
FEV_1,_% pred	75.2 ± 4.8	66.3 ± 2.8	60.1 ± 2.8	0.048	NS	0.023	NS
FEV_1_/VC %	61.5 ± 3.8	58.8 ± 2.0	54.4 ± 1.9	NS	NS	NS	NS
MEF_50,_ % pred	44.6 ± 10.2	34.5 ± 3.1	20.18 ± 2.1	< 0.0001	NS	< 0.0001	< 0.0001
CRP, mg/ml	4.4 ± 1.4	7.0 ± 0.9	8.9 ± 1.4	NS	NS	NS	NS

In the overall patients (Table 2), both PO and VO, as compared with no CVD, were associated with older age and lower VC, FEV_1_, FEV_1_/VC and MEF_50_ and higher CRP. The same associations (apart from CRP) were found in asthmatic patients (Table 3). In COPD patients (Table 4) the significant associations were with age and MEF_50,_ %.

The results of linear regression analysis are shown in Table 5 for the whole patients, in Table 6 for asthmatic patients and in Table 7 for COPD patients. In the overall patients predictive factors of CVD were: age, COPD, and sex; those of PO were COPD, BMI, VC, FEV_1_ and MEF_50_ and those of VO were age, class of disease severity, VC and MEF_50_. In asthma, the strongest predictors of CVD were VC, FEV_1_ and FEV_1_ /VC%, and PaO_2_, those of PO were VC, FEV_1_ and FEV_1_ /VC%; no predictor was found for VO. In COPD the predictors of CVD were age, GOLD class and sex; those of VO were age, VC and MEF_50_, while the only predictor of PO was BMI.

**Table 5:** Results of linear regression analysis for predictors of cardiovascular disease (CVD) in the overall patients

**Asthma + COPD**	**Non standardized coefficients**		**Standardized coefficient**	**t**	**Sig.**
	**B**	**SD Error**	**β**		
CVD					
Constant	.948	.248		3.829	.000
Age	.012	.003	.340	4.357	.000
COPD	.137	.063	.169	2.195	.030
Sex	.116	.058	.144	1.991	.048
VC, % pred	-.003	.002	-.124	−1.784	.076
MEF_50_, % pred	-.002	.001	-.131	−1.728	.086
Pressure overload					
Constant	1.066	.264		4.031	.000
COPD	.217	.074	.225	2.939	.004
BMI	.017	.007	.183	2.482	.014
VC, % pred	-.006	.003	-.213	−2.031	.044
FEV_1_, % pred	.007	.003	.322	2.162	.032
MEF_50_, % pred	-.006	.002	-.305	−2.668	.008
Volume Overload					
Constant	1.318	.446		2.954	.004
Age	.015	.004	.310	3.529	.001
GINA/GOLD class	-.118	.056	-.227	−2.113	.037
VC, % pred	-.006	.003	-.197	−2.244	.027
MEF_50_, % pred	-.005	.002	-.227	−2.094	.038

**Table 6:** Results of linear regression analysis for predictors of cardiovascular diseases (CVD) in asthma

**ASTHMA**	**Non standardized coefficients**		**Standardized coefficient**	**t**	**Sig.**
	**B**	**SD Error**	**β**		
CVD					
Constant	8.291	1.056		7.851	.000
Age	-.009	.005	-.270	−1.981	.059
BMI	.016	.008	.223	1.874	.073
VC, % pred	−.060	.009	−2.402	−6.630	.000
FEV_1_, % pred	.073	.011	3.667	6.894	.000
FEV_1_/VC, %	−.094	.013	−3.499	−7.021	.000
PaO_2_	−.010	.005	−.278	−2.268	.033
PRC	.016	.009	.209	1.797	.085
Pressure Overload					
Constant	6.668	1.082		6.163	.000
VC, % pred	−.060	.012	−1.874	−5.078	.000
FEV_1_, % pred	.067	.013	2.626	5.107	.000
FEV_1_/VC, %	−.077	.016	−2.237	−4.690	.000
Volume Overload					
Constant	−.116	.693		−.167	.869
Age	.019	.011	.320	1.822	.079

**Table 7:** Results of linear regression analysis for predictors of cardiovascular diseases (CVD) in COPD

**COPD**	**Non standardized coefficients**		**Standardized coefficient**	**t**	**Sig.**
	**B**	**SD Error**	**β**		
CVD					
Constant	.686	.393		1.747	.084
Age	.007	.003	.228	2.143	.035
GOLD Class	.076	.036	.241	2.089	.040
Sex	.121	.061	.203	1.983	.050
PaO_2_	.006	.003	.209	1.826	.071
Pressure Overload					
Constant	1.237	.229		5.414	.000
BMI	.020	.008	.244	2.337	.022
Volume Overload					
Constant	1.317	.519		2.539	.013
Age	.017	.005	.334	3.311	.001
VC, % pred	−.006	.003	−.220	−2.076	.041
MEF_50_, % pred	−.006	.003	−.305	−2.351	.021

As shown in Table 8, the predictors of IHD were COPD, age, BMI and FEV_1_ and those of PH, found only in COPD patients, were VO (present in 80% of the patients) and FEV_1_.

**Table 8:** Results of linear regression analysis for predictors of ischemic heart disease (IHD) and pulmonary hypertension (PH)

**IHD**	**Non standardized coefficients**		**Standardized coefficient**	**t**	**Sig.**
	**B**	**SD Error**	**β**		
IHD					
Constant	−1.486	.667		−2.229	.028
COPD	.450	.134	.412	3.351	.001
Age	.012	.004	.246	2.834	.005
BMI	.016	.008	.177	2.095	.038
FEV_1_, % pred	.007	.003	.309	2.127	.036
GOLD class	.150	.083	.294	1.810	.073
PH					
Constant	1.127	.244		4.613	.000
VO	.246	.045	.442	5.468	.000
GOLD class	−.105	.041	−.273	−2.584	.011
FEV_1_, % pred	−.004	.002	−.239	−2.222	.028

**Figure 1:**
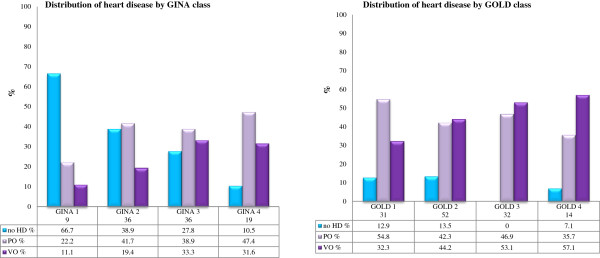
Distributions of heart pressure overload (PO) and volume overload (VO) by GINA asthma severity class (left graph), and by GOLD COPD severity class (right graph).

The distributions of PO and VO by severity of asthma (GINA) and COPD (GOLD) are reported in Figure 1. In asthma, there is a clear increase in the prevalence of both PO and VO with increasing the severity class, while in COPD the prevalence of each type of CVD is similar in all classes. The comparison between chronic bronchitis and emphysema patients, shown in Table 9, showed that the latter ones had older age, lower BMI, more severe airway obstruction and higher prevalence of heart volume overload and of pulmonary hypertension.

**Table 9:** Comparison between general characteristics of patients with chronic bronchitis and emphysema

	**Chronic bronchitis** **N. 102**	**Emphysema** **N. 27**	**P**
Age, years	68 ± 1.0	73 ± 1.5	0.017
BMI	27.2 ± 0.5	22.1 ± 0.6	< 0.0001
Male, n (%)	58 (56.9)	20 (74.1)	NS
Smokers			
current, n (%)	55 (53.9)	17 (63)	NS
past, n (%)	33 (32.4)	9 (33.3)	NS
Cardiovascular disease, n (%)	92 (90.2)	25 (92.6)	NS
Pressure overload,n (%)	51 (50)	8 (29.6)	NS
Volume overload, n (%)	41 (40.2)	17 (63)	0.034
Subgroup IHD n (%)	39 (39.4)	11 (40.7)	NS
Subgroup PH n (%)	15 (4.7)	10 (37)	0.009
GOLD class			
1	30 (29.4)	1 (3.7)	0.005
2	44 (43.1)	8 (29.6)	NS
3	19 (18.6)	13 (48.1)	0.0016
4	9 (8.8)	5 (18.5)	NS
VC, % predicted	82.7 ±2	85.5 ± 4.3	NS
FEV_1_, % predicted	68.1± 2.2	51.9 ± 3.1	0.001
FEV_1_/VC %	60.2 ± 1.4	46.56 ± 2.6	< 0.0001
MEF_50_, % predicted	33.7 ± 2.5	13.6 ± 2.1	< 0.0001
PaO_2_ , mmHg	71.2 ± 1.1	70.1 ± 2.1	NS
PaCO_2 ,_ mmHg	40.1 ± 0.6	39.0 ± 0.8	NS
CRP, mg/ml	7.9 ± 1.0	6.8 ± 1.2	NS

As one of the stronger predictors of CVD was age, we made a sub-analysis on subjects with an age equal to 65 years or higher. As shown in Table 10, the prevalence of CVD, PO and VO was similar in asthmatic and COPD patients; the latter had higher prevalence of men, smokers, lower FEV_1_, higher CRP and of IHD and PH. The results of linear regression analysis in these older patients are reported in Tables 11, 12 and 13.

**Table 10:** Comparisons between general characteristics of patients 65 years old or more

	**ASTHMA N. 33**	**COPD N. 92**	**P**
Age, years	71.8 ± 0.8	73.6 ± 0.6	NS
Male, n (%)	5 (15.2)	60 (65.2)	< 0.0001
Smokers			
Current n(%)	4 (13)	45 (51)	< 0.0001
Past n (%)	4 (13)	36 (41)	< 0.0001
BMI	25.8 ± 0.8	26.2 ± 0.5	NS
CVD, n (%)	30 (90.9)	87 (94.6)	NS
Pressure overload, n (%)	15 (45.5)	38 (41.3)	NS
Volume overload, n (%)	15 (45.5)	49 (53.3)	NS
Ischemic heart disease, n (%)	6 (18.2)	42 (46.7)	0.0054
Pulmonary hypertension, n (%)	0	21 (22.8)	0.0026
VC, % predicted	86.1 ± 2.6	82.7 ± 2.2	NS
FEV_1_, % predicted	73.3 ± 3.3	63.5 ± 2.3	0.023
FEV_1_/VC %	61.63 ± 2.5	56.4 ± 1.5	NS
MEF_50_, % predicted	30.8 ± 3.4	26.6 ± 2.3	NS
CRP, mg/l	4.1 ± 0.7	8.2 ± 1.0	0.018

**Table 11:** Results of linear regression analysis for predictors of cardiovascular disease in the overall patients 65 years old or more

**Asthma + COPD**	**Non standardized coefficients**		**Standardized coefficient**	**t**	**Sig.**
	**B**	**SD Error**	**β**		
CVD					
Constant	.879	.421		2.087	.040
Age	.020	.006	.380	3.581	.001
VC, % predicted	−.005	.002	−.308	−2.813	.006
CRP	−.006	.003	−.190	−1.707	.092
Pressure overload					
Constant	.895	.328		2.731	.008
Sex	.217	.094	.255	2.310	.024
BMI	.022	.011	.226	2.047	.044
Volume overload					
Constant	.293	.874		.335	.739
Age	.024	.011	.229	2.091	.040
VC, % predicted	−.006	.003	−.207	−1.883	.063

**Table 12:** Results of linear regression analysis for predictors of cardiovascular disease in asthmatic patients 65 years old or more

**Asthma**	**Non standardized coefficients**		**Standardized coefficient**	**t**	**Sig.**
	**B**	**SD Error**	**β**		
CVD					
Constant	7.203	.662		10.885	.000
VC, % pred	−.058	.007	−2.909	−8.345	.000
FEV_1_, % pred	.064	.008	4.069	7.728	.000
FEV_1_/VC, %	−.084	.011	−3.472	−7.509	.000
Pressure overload					
Constant	3.616	1.487		2.432	.035
Age	.028	.015	.250	1.913	.085
Sex	.864	.152	.864	5.691	.000
GINA class	−.380	.138	−.629	−2.766	.020
VC, % pred	−.032	.010	−1.209	−3.306	.008
FEV_1_, % pred	.033	.012	1.566	2.661	.024
FEV_1_/VC, %	−.043	.015	−1.333	−2.785	.019
PaO_2_	−.017	.006	−.392	−2.954	.014
Volume overload					
Constant	.293	.874		.335	.739
Age	.024	.011	.229	2.091	.040

**Table 13:** Results of linear regression analysis for predictors of cardiovascular disease in COPD patients 65 years old or more

**COPD**	**Non standardized coefficients**		**Standardized coefficient**	**t**	**Sig.**
	**B**	**SD Error**	**β**		
CVD					
Constant	.542	.464		1.169	.247
Age	.018	.006	.373	3.259	.002
BMI	.011	.006	.200	1.748	.085
VC,% predicted	−.003	.002	−.198	−1.724	.090
Pressure overload					
Constant	1.110	.330		3.366	.001
BMI	.025	.012	.263	2.060	.044
Volume overload					
Constant	.928	.480		1.932	.058
Sex	.384	.148	.341	2.598	.012
Smoking	.289	.112	.339	2.576	.013
VC, % predicted	−.006	.004	−.212	−1.763	.083

## Discussion

The results of this study show that patients with chronic airway obstructive disorders, either asthma or COPD, have increased prevalence of cardiovascular diseases as compared to the general population of similar age [18]. The comparison between the two disorders, (see Table 1) showed that CVD, particularly with volume overload, was more frequent in COPD patients, who had also older age, higher BMI, heavy smoking history, increased CRP and greater airway obstruction. This finding is in agreement with prior observations that in COPD comorbidities are frequent and are sustained by systemic inflammation and by smoking [4,6,7]. The comparison among patients without CVD, with PO and VO, showed that both pressure and volume overload were associated with older age and increased airway obstruction, either in asthma or in COPD, (see Tables 2, 3 and 4). These findings are in agreement with prior observations in COPD [8-13,19]. Unfortunately, relatively little research has been done in asthma [14]. It is generally believed that the link between asthma and CVD is less strong than that for COPD [14,20,21] and this applies particularly to ischemic heart disease in asthmatic males. By contrast, in women adult-onset asthma has been found to be a significant risk factor for IHD and stroke [21,22]. Although only 8 of our asthmatic patients had IHD, as many as 6 of them (75%) were never smoking women. However, the strongest predictors of IHD were COPD, age, BMI and FEV_1_, as previously suggested [19,23]. The strong association of COPD with IHD is attributed to the effect of cigarette smoking [19-24], and about 90% of our COPD patients were past or present smokers. The subanalysis of COPD patients with predominant chronic bronchitis or emphysema, (see Table 9), showed that the emphysema phenotype was associated with older age, lower BMI, more severe airway obstruction and higher prevalence of pulmonary hypertension and of volume overload. Unfortunately, there are no data in the literature comparing CVD in the two COPD phenotypes.

As mentioned above, age and COPD were the strongest predictors of CVD, but the two factors were closely linked each other, as COPD patients were significantly older than asthmatics. To clarify this point, we performed a sub-analysis on patients with an age equal to or higher than 65 years. As shown in Table 10, older asthmatics had the same prevalence of CVD than COPD patients (91% versus 95%). However, also in the elderly, the prevalence of IHD and PH was higher in COPD. Pulmonary hypertension deserves a special comment. PH in COPD is classically attributed to severe hypoxia causing raised pulmonary pressures, but other factors such as endothelial dysfunction or left heart disease, are deemed to play a role [24]. In our patients, the predictors of PH were FEV_1_ and volume overload, suggesting that both airway obstruction and post capillary mechanisms participated in increased pulmonary vascular resistance. Actually, VO was present in 80% of patients with PH.

## Conclusions

In conclusion, the results of this study indicate that cardiovascular disease are frequent in patients with chronic obstructive disorders, particularly COPD. The strongest predictors of CVD are age and severity of airway obstruction. In older patients the prevalence of CVD is similar in asthma and COPD, apart from ischemic heart disease and pulmonary hypertension, which are strongly associated with COPD.

## Abbreviations

COPD: Chronic obstructive pulmonary disease; CVD: Cardiovascular disease; FEV1: Forced expiratory volume in one second; IHD: Ischemic heart disease; MEF50: Maximum expiratory flow at mid VC; PH: Pulmonary hypertension; PO: Heart pressure overload; VO: Heart volume overload; VC: Vital capacity.

## Competing interests

The authors declare that they have no competing interests.

## Authors’ contributions

BC, BM and MM conceived and designed the study and contributed to drafting the manuscript, BM, MM, CA, CS, VA, BM contributed to acquisition and analysis of data, TR provided critical revision of the manuscript. All authors read and approved the final manuscript.
